# Cost-effectiveness of total knee arthroplasty, unicompartmental knee arthroplasty, and high tibial osteotomy for medial compartment knee osteoarthritis in young patients: a Canadian public payer perspective

**DOI:** 10.1186/s13018-025-05960-4

**Published:** 2025-05-31

**Authors:** Pakpoom Ruangsomboon, Onlak Ruangsomboon, Davis Tam, Bheeshma Ravi, Seper Ekhtiari, Daniel Pincus, Sebastian Tomescu

**Affiliations:** 1 Sunnybrook Holland Orthopaedic and Arthritic Centre, Division of Orthopaedic Surgery, University of Toronto, Toronto, ON Canada; 2 Sunnybrook Health Science Centre, University of Toronto, Toronto, ON Canada; 3 Faculty of Medicine, Siriraj Hospital, Mahidol University, Bangkok, Thailand; 4 Upstream Lab, Li Ka Shing Knowledge Institute, MAP Centre for Urban Health Solutions, Unity Health Toronto, Toronto, ON Canada; 5 Division of Orthopaedic Surgery, Department of Surgery, University of Toronto, Toronto, Canada; 6 Division of Orthopaedic Surgery, Department of Surgery, McMaster University, Hamilton, Canada; 7 Present address: Upstream Lab, MAP Center for Urban Health Solutions, St. Michael’s Hospital, 30 Bond St,, Toronto, ON M5B 1W8 Canada

**Keywords:** Cost-effectiveness, Cost-utility analysis, Unicompartmental knee arthroplasty, Total knee arthroplasty, High tibial osteotomy, Medial knee osteoarthritis, Markov model, Canada

## Abstract

**Background:**

In medial compartment osteoarthritis (OA) of the knee in young patients who fail conservative treatment, clinical equipoise exists between three surgical strategies: (1) total knee arthroplasty (TKA), (2) unicompartmental knee arthroplasty (UKA), and (3) medial opening wedge high tibial osteotomy (HTO). This study evaluated the cost-effectiveness of three surgical strategies, using a probabilistic Markov model from the Ontario public payer perspective in Canada.

**Methods:**

A probabilistic Markov model was developed to perform a cost-utility analysis comparing TKA, UKA, and HTO. The base case simulated a 45-year-old Canadian cohort with unilateral medial knee OA over a lifetime horizon. Outcomes included quality-adjusted life months (QALMs), discounted lifetime costs (1.5% annually), incremental cost-effectiveness ratios (ICERs), and net monetary benefit (NMB), reported in 2023 Canadian dollars (CAD, $). A willingness to pay (WTP) threshold of $4,166.67/QALM was applied. Model uncertainty was assessed via 3,000 iterations of probabilistic sensitivity analysis. Scenario analyses using sex-specific mortality rates were also conducted.

**Results:**

Mean costs were $9,157 (TKA), $9,238 (HTO), and $9,419 (UKA). UKA produced the highest QALMs (290.53), followed by TKA (277.02) and HTO (270.88). HTO was absolutely dominated, as it was both more costly and less effective than TKA. Among undominated strategies, UKA yielded an ICER of $19.46/QALM compared to TKA. UKA also had the highest NMB ($1,201,112), outperforming TKA ($1,145,110) and HTO ($1,119,411). UKA was the most cost-effective option in 55.27% of probabilistic simulations, followed by TKA (23.83%) and HTO (20.90%). Scenario analyses with sex-specific mortality showed similar trends.

**Conclusions:**

UKA is the most cost-effective surgical strategy from a public payer perspective for young patients with medial knee OA. At a WTP of $4,166.67/QALM, UKA balances long-term durability and economic value better than TKA or HTO.

**Level of evidence:**

Level III, Model-based economic evaluation.

**Supplementary Information:**

The online version contains supplementary material available at 10.1186/s13018-025-05960-4.

## Introduction

Isolated medial compartment knee osteoarthritis (OA) commonly affects younger adults, especially around the age of 45, due to prior trauma, malalignment, or early degeneration [1, 2]. While non-operative therapies may help early on, many patients eventually require surgery due to pain and functional decline [3, 4]. In patients who fail conservative treatment, clinical equipoise exists among three surgical strategies: total knee arthroplasty (TKA), unicompartmental knee arthroplasty (UKA), and medial opening wedge high tibial osteotomy (HTO) [5–7]. Each has distinct benefits and risks: TKA offers durable outcomes but is more invasive; UKA, by contrast, has the ability to resurface only the medial compartment, preserves native anatomy and may better suit younger active patients [7–9]; and HTO provides joint preservation via mechanical axis correction with favorable outcomes in selected younger patients [10–12].

Importantly, TKA, UKA, and HTO differ not only in invasiveness and indications, but also in their failure patterns—particularly the risk of revision due to knee instability versus mechanical failure without instability. Revision risk due to ligament instability after UKA is the lowest compared to HTO and TKA [13–16], likely due to a minimally invasive exposure that preserves the native ligaments and restores pre-arthritic knee biomechanics. In contrast, HTO alters the mechanical axis and may require partial release of the medial collateral ligament [12], while TKA often involves soft tissue balancing or ligament releases to accommodate the prosthesis, potentially increasing the risk of postoperative instability [13–16]. These variations influence revision strategies, long-term outcomes, and economic consequences. However, no existing cost-effectiveness analyses have incorporated these differential failure modes into a lifetime Markov model, especially for a young adult cohort under a publicly funded healthcare system.

To address this gap, we developed a probabilistic Markov model simulating the cost-utility of TKA, UKA, and HTO in 45-year-old patients with medial compartment OA over a lifetime horizon. The objective was to identify the most cost-effective strategy under a Canadian public payer perspective.

## Methods

### Study design and setting

We developed a probabilistic Markov cohort model using TreeAge Pro 2025 (TreeAge Software Inc., Healthcare version R1.0, Williamstown, MA) to perform a cost-utility analysis (CUA) from the perspective of the Ontario Ministry of Health and Long-Term Care—a public third-party healthcare payer. The model estimated lifetime clinical outcomes, including quality-adjusted life expectancy (reported in quality-adjusted life months [QALMs]), total lifetime costs, net monetary benefit (NMB), and incremental cost-effectiveness ratios (ICERs) between competing surgical strategies [17, 18].

### Data sources

We collected data from various sources, including the patient-level direct and indirect medical costs from the case costing unit of Sunnybrook Holland Orthopeadic and Arthritic Centre Hospital, the largest volume arthroplasty center in Canada, for the fiscal year 2023–2024; the Canadian Joint Replacement Registry (CJRR), population-based data [16]; and relevant literature [16, 19–24]. We utilized data specific to Ontario, the most populated province in Canada, whenever feasible. The medical costs obtained from the hospital case costing unit included a comprehensive set of healthcare expenses, encompassing inpatient and perioperative care. These data incorporated costs for patient food, acute nursing care, operating room expenses, emergency department visits, diagnostic and therapeutic laboratory tests, respiratory therapy, pharmacy, clinical nutrition, physiotherapy, occupational therapy, speech-language therapy, social worker consultations, recreational therapy, intensive care unit stays, and recovery room utilization. Surgical care costs included day surgery, pre- and post-operative care, as well as costs related to perioperative interventions, such as operating room and post-anesthesia recovery. Physician costs for each procedure were derived from the Ontario Schedule of Benefits [25]. Probabilities of events at each Markov stage were applied based on population-level literature focusing on outcomes for young patients with medial compartment knee OA undergoing surgery after failed conservative treatment [9, 13–16, 19, 20, 23, 24, 26, 27]. The population mortality rate for the simulated cohort was incorporated using data from Statistics Canada, and relevant literature [16, 22, 24, 26]. We applied an annual discount rate of 1.5% to both QALMs and costs based on the current Canadian Agency for Drugs and Technologies in Health (CADTH) guidelines [28]. We used within-cycle correction to compensate for biases occurring with discrete-time rather than continuous-time health state transitions [29]. Reporting of results follows the CHEERS 2022 guidelines (Supplementary Table 1) [30].

### Base case cohort

We simulated a cohort of 45-year-old Canadian patients with unilateral medial compartment knee OA who were candidates for surgery after failed non-operative treatment. Age 45 was selected to reflect the common clinical scenario of younger patients facing surgical equipoise with a long potential duration for implant survivorship, different types of subsequent surgery, and health utility gains [1, 2, 31].

### Model type and structure

We constructed a discrete-time Markov cohort model to evaluate three surgical strategies: TKA, UKA, and HTO. Time was modelled in uniform, discrete time steps of one month duration. The one-month cycle allowed sufficient granularity to capture perioperative events and complications.

The simulation cohort entered the Markov model, underwent one of the treatment strategies, and then experienced subsequent events, such as revision, which were modelled stochastically [32]. Subsequent to the index surgery, the cohort entered a collection of mutually exclusive health states. During a time step, the portion of the cohort occupying a particular state could remain in that state or transition to another according to transitional probabilities. At each time step, the sum of discounted QALMs and discounted costs across the states were added to a cumulative total. At each step, there was a probability of transition to the death state such that, eventually, the majority of the cohort had died, the cumulative total QALMs and costs asymptotically approached their final values, and the simulation was terminated. The modelled events after surgery were modelled as being stochastic and included perioperative death, entry into the well-after-the-primary-operation state, or transition to a treatment failure state after the initial strategy. The probability of treatment failure was modelled to be different for each surgical intervention.

**Fig. 1:**
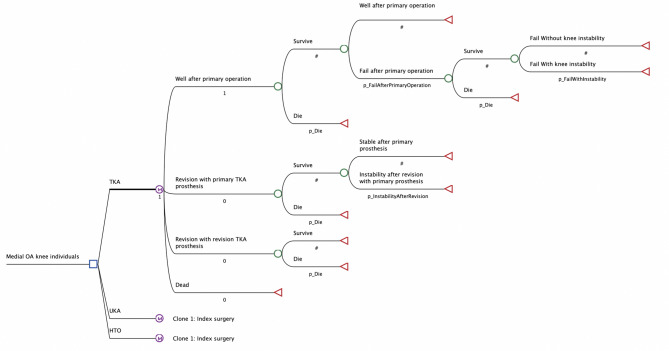
Markov model structure comparing three surgical strategies—TKA, UKA, and HTO—for patients with isolated medial compartment knee osteoarthritis (OA) following failed conservative treatment. Abbreviations: TKA– Total Knee Arthroplasty, UKA– Unicompartmental Knee Arthroplasty, HTO– medial opening wedge High Tibial Osteotomy

Figure 1 depicts an overview of the model structure and possible transitions among these health states in subsequent Markov cycles. At the beginning of the first cycle, a simulated cohort could only be in one of the following: (1) TKA, (2) UKA, and (3) HTO. In subsequent model cycles, a simulated cohort could remain in the same health state or transition to another. The cycles continued until all cohort transitioned to “Dead.”. The model simulation was defined to terminate at 540 months (equivalent to age 90), based on mortality trends from the general Canadian population, which indicate that most individuals would be less likely to survive beyond this time frame [26].

### Treatment strategies

#### Arthroplasty options (TKA and UKA)

Simulated patients who underwent either TKA or UKA could experience perioperative mortality, treatment success, or treatment failure. Treatment failure was categorized as occurring with or without postoperative joint instability. The model structure explicitly captures the unique trajectories of post-revision events. The type and cause of revision—particularly knee failure with instability—differ between TKA, UKA, and primary TKA after HTO, and these were modeled accordingly. The TKA pathway following a failed HTO accounted for the increased surgical complexity and risk of ligamentous insufficiency, which may necessitate the use of high-stability implants and influence the probability of revision. Knee instability-related failure required revision surgery, and in the case of recurrent instability, patients would undergo revision using high-stability prosthesis (varus-valgus constraint, VVC). Treatment failure without instability was modeled as requiring primary revision surgery using non-VVC type prostheses (first revision). After the first revision, patients entered a post-revision health state, with the potential for either staying well after recovery or further experiencing treatment failure. In cases of repeated failure, a second revision was modeled with VVC prostheses. Beyond the second revision, no further surgical intervention state was constructed, and patients remained in the post-revision state until death or simulation reached the termination condition.

#### Medial opening wedge HTO

Patients undergoing HTO could stay well after the index surgery, experience perioperative complications, perioperative death, or progressive symptoms leading to TKA after HTO. When subsequent surgery occurred, patients entered the TKA pathway with subsequent transitions to revision states as outlined earlier. The pathway of TKA after HTO followed the same structure as that of primary TKA.

#### Other transitional probabilities and mortality

At each chance node (a decision point where events occur based on probability, such as complication or revision), patients transitioned between health states according to probability estimates derived from published studies. Transitional probabilities of complications were derived from large registries, including CJRR database, and meta-analyses reporting composite failure outcomes [13–16]. These sources inherently incorporate the cumulative effects of perioperative and longer-term complications, including infection, fracture, and instability. As such, although individual perioperative complications were not modeled as separate short-term health states, their effects are captured within the overall failure probabilities applied in the model. Survivorship data were converted into transitional probabilities using the exponential function (S(t) = exp[–rate × t]), which allows for appropriate incorporation of observed failure risk into cycle-based transitions. Meanwhile, mortality was dynamically modeled to increase with age, using annual updates from Canadian life Table [26]. The simulation continued until simulated patients transitioned to the death state or reached the model’s termination condition.

#### Input parameters

Baseline probabilities, utilities, and costs were sourced from systematic reviews, clinical trials, and large observational studies as detailed in the ‘Data sources’ section. Parameter values, standard deviations (SD), and applied distribution types are provided in Table 1. For studies that did not report standard deviations, we estimated SD as 20% of the mean [33].

**Table 1:** Base case parameter estimates for probabilities, utilities, and costs

Parameter	Base case	Plausible range	Sources
**Probability of events (per month)**	Value		
TKA failure after index surgery (start age 45 years) UKA failure after index surgery HTO failure after index surgery TKA failure with instability after index surgery UKA failure with instability after index surgery HTO failure with instability after index surgery Canadian general population mortality during age 45–90 Canadian male mortality during age 45–90 Canadian female mortality during age 45–90	29.559 × 10^− 4^ 8.1627 × 10^− 4^ 33.158 × 10^− 4^ 0.124 0.062 0.095 * * *	23.946–36.587 × 10^− 4^ 7.433–8.897 × 10^− 4^ 32.449–46.393 × 10^− 4^ 0.099–0.149 0.049–0.074 0.076–0.114 * * *	[9,31] [9,20] [27] [20] [17] [19] [30] [30] [30]
**Utilities (two-parameter beta distribution)**	Mean (SD)		
Staying well after TKA Staying well after UKA Staying well after HTO Fail TKA without instability Fail UKA without instability Fail HTO without instability Fail index surgery with knee instability Staying well after revision by primary TKA Staying well after TKA in prior HTO Staying well after revision by high stability implant Discount rate	0.840 (0.150) 0.870 (0.130) 0.835 (0.167) 0.610 (0.200) 0.690 (0.140) 0.690 (0.140) 0.500 (0.300) 0.772 (0.154) 0.804 (0.161) 0.740 (0.180) 1.5%	0–1 0–1 0–1 0–1 0–1 0–1 0–1 0–1 0–1 0–1 N/A	[23] [23] [24] [23] [23] [24] [24] [24] [24] [23] [32]
**Costs (gamma distribution)**,** CAD**	Mean (SD)		
**Provider fees** Estimated physician cost for TKA Estimated physician cost for TKA with hardware removal Estimated physician cost for UKA Estimated physician cost for HTO Estimated physician cost for revision TKA Total costs** (excluding provider costs) Primary TKA with/without hardware removal Primary UKA Primary HTO Revision using primary TKA prosthesis Revision using higher constraint TKA prosthesis	$1,146 (229) $1,299 (259) $1,067 (213) $950 (190) $1,547 (309) $8,010 (1,738) $8,272 (738) $8,091 (2,929) $8,178 (1,636) $14,739 (2,948)	0–1,548 0–1,752 0–1,440 0–1,283 0–2,088 0–11,067 0–9,523 0–13,433 0–11,041 0–19,899	[29] [29] [29] [29] [29] Patient-level data Patient-level data Patient-level data Patient-level data Patient-level data

* See Supplementary Table 2 for full details of mortality rate conversion per one month cycle for general population, males, and females

** Including costs for prosthesis, operating room, pharmacy, day surgery pre- and post-operative care, recovery room, patient food, acute nursing, diagnostic and therapeutic lab, respiratory therapy, clinical nutrition, physiotherapy, occupational therapy, speech language, social working, and recreation therapy costs

Abbreviations: TKA– Total Knee Arthroplasty, UKA– Unicompartmental Knee Arthroplasty, HTO– medial opening wedge High Tibial Osteotomy, SD– Standard Deviation, CAD, $– Canadian Dollars

#### Model assumptions

Several assumptions were applied. *Markovian assumption -* The model followed the Markovian assumption, in which each transition depended only on the current health state and not on prior events [17]. *Homogeneity assumption* - As a cohort, patient heterogeneity (e.g., specific comorbidities, deformity, anterior cruciate ligament status) were not considered. However, given the focus on relatively young adults, it was assumed that patients in this demographic are generally healthier and less likely to have severe comorbidities that would significantly affect inpatient costs or postoperative outcomes. *Physician billing costs -* For physician costs, we used only the surgeon’s orthopaedic billing code from the Ontario Schedule of Benefits for each surgical procedure [25]. Other physician-related billing codes—such as those for anesthesiologists, surgical assistants, or consultants—were not explicitly modeled and assumed to be similar across strategies. *Infection*– septic failures were not modeled as separate states, based on evidence showing similar infection rates between UKA, TKA and HTO [34]. *Revision implants for failure with instability*– All instability failtures were treated with VVC prostheses. We did not capture the cost or outcome differences associated with the use of hinged knee prostheses, which are typically reserved for global ligamentous insufficiency [35].

#### Model validation

We evaluated the face validity of the model structure and outputs by consensus among five high-volume arthroplasty surgeons (PR, BR, SE, DP) and a joint preservation specialist surgeon (ST), ensuring the model structure and outputs were consistent with clinical expectations. Internal and external model validation were also conducted by an internal check of the model’s outputs against the prevalence of revision surgery and mortality rates post-surgery (Supplementary Fig. 1–3).

### Cost-utility analysis and statistical methods

Model simulation estimated total costs (C) and effectiveness (E), expressed in QALMs, for all three treatment strategies. With 3,000 iterations, these values were then averaged across iterations to generate expected costs and outcomes per strategy. A strategy was classified as “dominated” if it was both more costly and less effective than an alternative [36], and was subsequently excluded from further comparisons. To compare undominated strategies, we computed the ICER by dividing the difference in costs (ΔC) by the difference in effectiveness (ΔE) between two strategies using the formula: ICER = ΔC/ΔE [18]. While ICER is widely recognized, it is prone to interpretive challenges in multi-strategy comparisons. NMB and incremental NMB (INMB) offer more stable, linear comparisons [17, 36]. The NMB for each strategy was calculated using the formula: NMB = (E × WTP)– C [36], where WTP refers to the willingness-to-pay threshold, set at $4,166.67 CAD/QALM (equivalent to $50,000 CAD/quality-adjusted life year [QALY]) [37, 38]. A higher NMB reflects a more economically attractive strategy. INMB represents the monetary gain of one strategy over another at a given WTP, with a positive INMB indicating greater cost-effectiveness.

To evaluate uncertainty of the model findings on different WTP thresholds, we constructed a cost-effectiveness acceptability curve (CEAC) by varying the WTP values and calculating the probability of being the cost-effective option for each strategy [18].

### Sensitivity analyses

#### Deterministic sensitivity analyses (DSA)

To evaluate the robustness of the results, we performed one-way and two-way DSA to assess the effect of model parameter uncertainty. For one-way DSA, we ran the model at pre-specified intervals for each included variable within its plausible range while keeping all other variables fixed at the mean value of their distribution. We considered the model robust to a variable if the overall results (i.e., favourable treatment strategy) did not change from the main analysis. We reported one-way DSA by the Tornado diagram to display the parameters with the greatest impact on the NMB results between two undominated strategies. Consequently, we employed a two-way DSA to explore the combined effect of the two most influential parameters from the Tornado diagram findings.

#### Probabilistic sensitivity analysis (PSA)

We then performed a second-order Monte Carlo simulation to assess how parameter uncertainty influenced model outcomes. We assigned probability distributions to each parameter to reflect the uncertainty around their point estimates and associated 95% confidence intervals. For each of the 3,000 iterations, a random set of values was drawn from these distributions and used to run the model. We demonstrated a kernel probability density plot of the NMB distribution for each strategy across 3,000 PSA iterations. We applied gamma distributions for costs and two-parameter beta distributions for utility values [18].

#### Scenario analysis

We examined whether sex-specific differences in life expectancy would affect model outcomes. To adjust for sex-specific mortality, we conducted a scenario analysis using mortality rates stratified by sex (male vs. female). While the base case used age-adjusted general population mortality, the scenario analysis applied sex-specific annual mortality probabilities from Statistics Canada life Table [26]. The model structure and assumptions remained the same, except for the dynamic change of sex-specific mortality rates after one year cycle.

## Results

### Base case analysis

#### Costs and QALMs of each strategy

Among the three strategies, TKA had the lowest mean cost at $9,156.68 CAD, followed by HTO at $9,238.38 CAD. UKA was the most expensive at $9,419.39 CAD (Table 2). The discounted average QALMs for UKA yielded the highest mean QALMs at 290.53, followed by TKA at 277.02 QALMs. HTO provided the lowest benefit gained among the three strategies, with 270.88 QALMs (Table 2).

**Table 2:** League table results of the base case and scenario analyses

Dominance	Strategy	Cost (CAD)	Incr Cost (CAD)	Eff (QALMs)	Incr Eff (QALM)	ICER (CAD/ QALM)	NMB (CAD)*
**Base case analysis**							
Undominated	TKA	9,156.68	-	277.02	-	-	1,145,110.43
Absolutely dominated	HTO	9,238.38	81.70	270.88	-6.15	Dominated	1,119,410.67
Undominated	UKA	9,419.39	262.71	290.53	13.50	19.46	1,201,111.75
**Scenario analysis - Male cohort**							
Undominated	TKA	9,156.68	-	268.55	-	-	1,109,828.70
Undominated	UKA	9,419.39	262.71	281.54	12.99	20.22	1,163,994.64
**Scenario analysis - Female cohort**							
Undominated	TKA	9,156.68	-	285.27	-	-	1,178,787.76
Undominated	UKA	9,419.39	262.71	299.27	14.00	18.76	1,236,378.99

Abbreviations: Incr Cost– Incremental Cost (difference in cost between strategies), Eff– Effectiveness (measured in QALMs), QALMs– Quality-Adjusted Life Months, CAD– Canadian Dollars, Incr Eff– Incremental Effectiveness (difference in effectiveness between strategies), ICER– Incremental Cost-Effectiveness Ratio, NMB– Net Monetary Benefit, TKA– Total Knee Arthroplasty, HTO– medial opening wedge High Tibial Osteotomy, UKA– Unicompartmental Knee Arthroplasty,

Note: * NMB = (QALE x willingness to pay[WTP])– Cost; WTP = 4,166.67 CAD/QALM

**Fig. 2:**
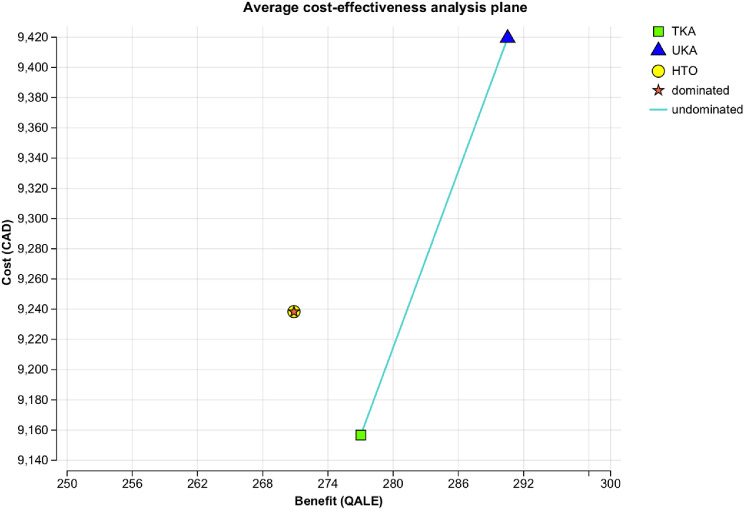
Average cost-effectiveness analysis plane. Abbreviations: TKA– Total Knee Arthroplasty, UKA– Unicompartmental Knee Arthroplasty, HTO– medial opening wedge High Tibial Osteotomy, CAD, $– Canadian Dollars; QALE– Quality-adjusted Life Expectancy

Figure 2 shows an average cost-effectiveness analysis plane, illustrating the relationship among the three strategies. HTO was absolutely dominated, meaning it was both more costly and less effective than TKA. As a result, HTO was excluded from subsequent comparisons.

#### ICER

After removing the dominated option, the ICER comparing UKA to TKA was $19.46 CAD/QALM, indicating that UKA is cost-effective relative to TKA under the WTP threshold of $4,166.67 CAD/QALM (Table 2).

#### NMB and INMB

At the WTP of $4,166.67 CAD/QALM, UKA had the highest NMB at $1,201,111.75 CAD, followed by TKA at $1,145,110.43 CAD, and HTO at $1,119,410.67 CAD (Table 2). After excluding HTO, the INMB for UKA vs. TKA was +$56,001.32 CAD, confirming UKA as the more cost-effective option at this WTP.

#### Model uncertainty

**Fig. 3:**
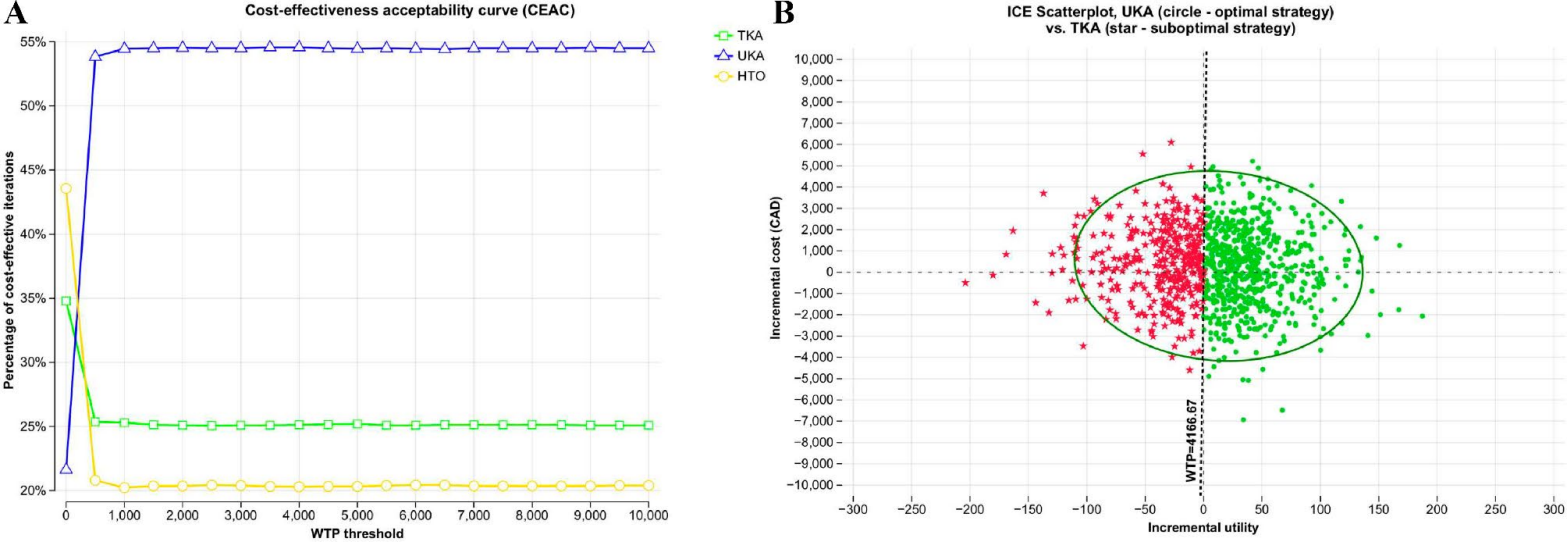
**A**. Cost-effectiveness acceptability curve (CEAC). Abbreviations: TKA– Total Knee Arthroplasty, UKA– Unicompartmental Knee Arthroplasty, HTO– medial opening wedge High Tibial Osteotomy, NMB– net monetary benefit, WTP– willingness to pay. **B**. Incremental cost-effectiveness scatter plot between TKA and UKA. Abbreviations: TKA– Total Knee Arthroplasty, UKA– Unicompartmental Knee Arthroplasty, CAD, $– Canadian Dollars, WTP– willingness to pay

Based on the CEAC (Fig. 3A), at WTP = $0 CAD/QALM (not willing to pay any dollar for QALM), HTO and TKA were initially more attractive, with UKA having only a 22% probability of being optimal. However, once WTP exceeded $500 CAD/QALM, UKA became the most cost-effectiveness strategy, maintaining a stable probability of 55% across higher thresholds. At WTP = $4,166.67 CAD/QALM, most simulations favored UKA, falling predominantly within the northeast quadrant below the WTP line in an incremental cost-effectiveness (ICE) plane (Fig. 3B), thus supporting the dominance of UKA over TKA.

### Sensitivity analyses

#### One-way and two-way DSA

**Fig. 4:**
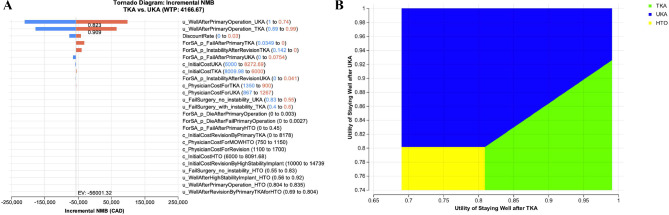
**A**. Tornado diagram showing one-way deterministic sensitivity analysis results. Abbreviations: TKA– Total Knee Arthroplasty, UKA– Unicompartmental Knee Arthroplasty, HTO– medial opening wedge High Tibial Osteotomy, NMB– net monetary benefit, CAD, $– Canadian Dollars, WTP– willingness to pay. **B**. Two-way deterministic sensitivity analysis results showing the optimal strategy under different utilities of staying well after TKA and UKA Abbreviations: TKA– Total Knee Arthroplasty, UKA– Unicompartmental Knee Arthroplasty, HTO– medial opening wedge High Tibial Osteotomy

The tornado diagram (Fig. 4A) shows that postoperative utility after UKA was the most influential parameter. UKA lost its dominance when this utility fell below 0.823. Other key drivers included post-TKA utility, discount rate, and revision probabilities for UKA and TKA. Two-way DSA explored the optimal strategy under different utilities of staying well after TKA and UKA at WTP of $4,166.67 CAD/QALM (Fig. 4B). It revealed that UKA remained the optimal strategy when its utility exceeded 0.82, particularly when TKA utility was moderate to low. TKA became favorable only when its utility exceeded 0.90 and UKA utility dropped. HTO became optimal only under extreme conditions when both UKA and TKA utilities were below 0.80.

#### PSA

**Fig. 5:**
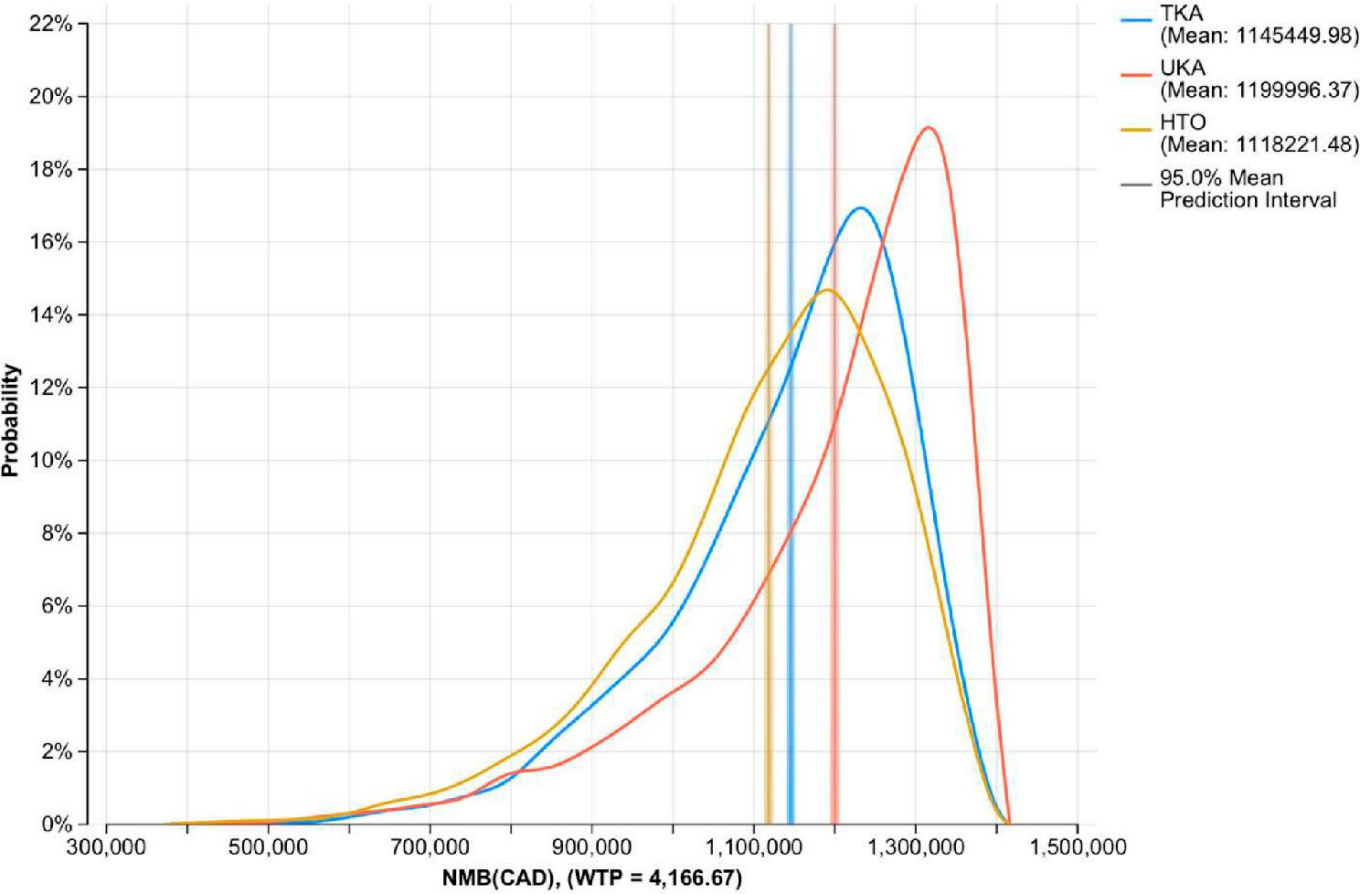
NMB kernel probability density plot for the base case analysis. Abbreviations: TKA– Total Knee Arthroplasty, UKA– Unicompartmental Knee Arthroplasty, HTO– medial opening wedge High Tibial Osteotomy, NMB– net monetary benefit, CAD, $– Canadian Dollars, WTP– willingness to pay

Across 3,000 iterations, UKA had the highest mean NMB at $1.20 M CAD, followed by TKA ($1.15 M) and HTO ($1.12 M). UKA was the most cost-effective in 55.27% of the simulations, compared to 23.83% for TKA and 20.90% for HTO. The Kernel probability density plot of the NMB distribution across 3,000 iterations is shown in Fig. 5. UKA consistently yielded the highest NMB, with a peak density shifted rightward compared to both TKA and HTO, indicating greater economic value. While there is an overlap among the curves, UKA shows a tighter and more right-skewed distribution, reflecting more frequent higher NMB outcomes. TKA follows closely but with a broader distribution and lower peak. HTO consistently displayed the lowest NMB, with its curve concentrated further to the left, confirming its inferior economic performance relative to UKA and TKA.

### Scenario analysis

Results of the scenario analysis were consistent with those of the base case (Table 2). In the male-only scenario, UKA cost $9,419.39 CAD and yielded 281.54 QALMs, with a 54.47% probability of being cost-effective. TKA followed with $9,156.68 CAD and 268.55 QALMs (23.57% probability). HTO remained absolutely dominated. For the female-only cohort, UKA yielded 299.27 QALMs at $9,419.39 CAD and was cost-effective in 53.57% of the simulations. TKA provided 285.27 QALMs at $9,156.68 CAD with a 25.40% probability of being optimal. HTO was absolutely dominated.

## Discussion

This study assessed the cost-effectiveness of three surgical strategies for medial compartment knee OA in younger patients from a public payer perspective in Ontario, Canada. HTO was absolutely dominated—being more costly and providing lower utility gained than TKA— and was thus excluded from further comparisons. Among the remaining undominated options, UKA yielded greater QALMs at a slightly higher cost than TKA, resulting in a favorable ICER. Both NMB and INMB confirmed UKA as the most economically attractive strategy at a WTP threshold of $4,166.67 CAD/QALM. The CEAC demonstrated that UKA consistently had the highest probability of being cost-effective across a wide range of WTP values. UKA only lost dominance under unrealistically low WTP scenarios (e.g., < $600 CAD/QALM), which are unlikely within Canada’s public healthcare system [28]. Scenario analyses using sex-specific mortality rates yielded the same trend, supporting the base case findings.

To our knowledge, this study presents the first lifetime CUA comparing UKA, TKA, and HTO in young patients with medial knee OA. Our model uniquely incorporated distinct modes of failure—including instability-related and non-instability-related failures—which influence the type and cost of revision procedures. These nuances have not been explicitly captured in previous economic evaluation studies [20, 21, 39]. By addressing this gap, our study not only reflects current clinical complexities but also enhances decision-making relevant for public payers. The findings align with recent registry data and emerging clinical literature, which showed favorable outcomes and increasing use of UKA in younger, active patients [40, 41].

While UKA is relatively more expensive and requires careful patient selection criterias to achieve favorable results [21, 42, 43], it offers several advantages for younger, active patients, including the preservation of native knee mechanics and cruciate ligaments, and faster recovery. It has also demonstrated superior kinematic restoration compared to TKA [44–47]. TKA, though more invasive, remains a reliable option with excellent long-term survivorship [9], particularly for patients with more advanced deformities or those not eligible for UKA [48]. In our model, TKA was the least costly strategy but yielded fewer QALMs than UKA, resulting in a lower NMB. Medial opening wedge HTO is typically recommended for younger, high-demand individuals, while being less suitable for older patients due to a higher risk of failure [12]. However, in this analysis, HTO was classified as a dominated strategy. This may be due to its longer initial recovery period associated with restricted weight-bearing, which reduces short-term utility gains, as well as the risk of progressive osteoarthritis requiring later conversion to TKA [49–51]. These factors cumulatively lowered its lifetime cost-effectiveness within the model.

Nevertheless, despite the favorable cost-effectiveness profile of UKA in our model, its adoption in Ontario remains relatively low. A population-based study by Ekhtiari et al. identified only 4,385 UKA procedures performed between 2002 and 2006 across the province, highlighting its underutilization compared to TKA despite a 10-year survivorship exceeding 80% and no significant differences in revision risk across socioeconomic strata [52]. These favorable outcomes support the real-world feasibility and equity of UKA in appropriately selected patients. Our findings align with this evidence and may help inform policymakers and funding agencies aiming to optimize the value-based delivery of arthroplasty care within the public system.

Our findings offer different yet legitimate perspectives compared to prior economic evaluations, such as those by Slover et al. and Konopka et al. [20, 39], which assessed different patient populations and were under different model assumptions. Slover et al. [39] focused on elderly, low-demand patients and identified UKA as cost-effective only under limited revision risk, whereas our model targeted younger adults and accounted for a broader lifetime horizon. Konopka et al. [20] emphasized that the cost-effectiveness of UKA and HTO depended heavily on conversion rates to TKA and outcomes following conversion. Similarly, Kazarian et al. used a Markov model to assess UKA, TKA, and non-surgical treatment, showing UKA to be cost-effective across all ages [53]. In contrast, our model was structured to distinguish between failure modes—particularly instability-related failure versus non-instability-related failure—and their implications on revision implant type and cost. In addition, prior analyses included a broader age range and/or excluded HTO, whereas our study focused specifically on younger cohort, who are candidates facing surgical equipoise between all three surgical options. This refinement reflects real-world surgical practice where TKA after HTO can often proceed using standard primary implants rather than costly revision components, diminishing the huge impact of conversion rates on the overall cost-effectiveness. Notably, our Tornado diagram showed that utilities for well states after UKA and TKA were the primary drivers of model outcomes, while failure probabilities had comparatively less influence. This finding suggests that patient-reported outcomes may play a more pivotal role in long-term cost-effectiveness than revision incidence alone.

While our findings suggest that UKA is the most cost-effective option among the three surgical strategies, this conclusion assumes clinical equipoise—that patients are equally eligible for TKA, UKA, or HTO. In reality, surgical candidacy is determined by several factors that fall outside the scope of this economic model, including the degree of preoperative deformity, ligamentous integrity (e.g., anterior cruciate ligament deficiency), and patient-specific goals or activity levels. For instance, patients with moderate arthritis or those with uncorrectable deformities may not be ideal candidates for UKA despite its favorable cost-effectiveness. Therefore, while this study offers important economic insights, clinical decision-making must remain individualized, guided by patient anatomy, functional demands, and surgical feasibility.

### Strengths and limitations

This study has several strengths. First, the model incorporated realistic revision pathways, including instability-related failure and subsequent high-stability implant usage. Second, dynamic age-based mortality updates based on national life tables allowed us to reflect survival changes over the patient’s lifespan. Third, our simulation included post-failure transitions and conversions, ensuring clinical plausibility. Fourth, we used actual hospital case costing data and CJRR-based procedure profiles, which increase the generalizability and relevance to the Canadian public system. Lastly, our probabilistic model provides a comprehensive exploration of uncertainty, which strengthens the robustness of our conclusions.

Nonetheless, this study has several limitations. First, as a Markov cohort model, individual patient-level characteristics (e.g., age variation, sex, comorbidities) were not explicitly modeled due to the inherent assumption of homogeneity in the cohort [18]. Future research using microsimulation or two-dimensional Monte Carlo model could address this limitation by capturing patient heterogeneity [17, 32]. Second, only the primary orthopedic billing code was included for provider costing, while anesthesia and assistant fees were not. These were assumed to be comparable across strategies and were not found to significantly influence model outcomes, as demonstrated in the Tornado diagram. Third, the model did not separately account for periprosthetic joint infection or osteotomy-related infection. However, a recent systematic review found no significant differences in infection rates across TKA, UKA, and HTO [34], suggesting minimal bias from this omission. Fourth, robotic-assisted arthroplasty was not included as a comparator due to its limited availability across public centers in Ontario and the distinct perioperative costs and revision trajectories that would necessitate a separate modeling framework. While robotic-assisted surgery has not demonstrated definitive superiority in reducing long-term complications or improving clinical outcomes over conventional arthroplasty [54, 55], their adoption is increasing globally. We acknowledge the relevance of this trend and emphasize the importance of future cost-effectiveness analyses specifically evaluating robotic-assisted approaches in younger, active patients. Lastly, our findings were based on a base case cohort of 45-year-old Canadian patients and may not be generalizable to older populations or other healthcare systems with different WTP thresholds.

## Conclusion

UKA is the most cost-effective surgical strategy for treating medial compartment OA of the knee in young patients from Canada’s healthcare payer perspective. At a WTP threshold of $4,166.67 CAD/QALM, UKA consistently outperformed both TKA and HTO. Our findings support a broader adoption of UKA in appropriately selected younger patients, and provide key evidence to guide clinical decision-making and healthcare policy.

## Electronic supplementary material

Below is the link to the electronic supplementary material.

Supplementary Material 1

Supplementary Material 2

Supplementary Material 3

## Data Availability

Deidentified individual-level data is kept at the hospital database and will not be shared publicly. All other model inputs are publicly available, and the complete model is accessible at GitHub repository - https://github.com/PakpoomRuangsomboon/MedialOAknee_CUA_Ontario-Model-PR.

## Declarations

### Statement of location

Our work was performed in five institutions; (1) Sunnybrook Health Sciences Centre, University of Toronto, Toronto, Ontario, Canada (2) Faculty of Medicine, Siriraj Hospital, Mahidol University, Thailand (3) Upstream Lab, MAP Centre for Urban Health Solutions, Li Ka Shing Knowledge Institute, Unity Health Toronto, Toronto, Ontario, Canada (4) ICES, Toronto, Canada. 5) McMaster University, Hamilton, Canada.

## Abbreviations

OA: Osteoarthritis
TKA: Total Knee Arthroplasty
UKA: Unicompartmental Knee Arthroplasty
HTO: Medial Opening Wedge High Tibial Osteotomy
QALMs: Quality-Adjusted Life Months
ICERs: Incremental Cost-Effectiveness Ratios
NMB: Net Monetary Benefit
CAD, $: Canadian dollars
WTP: Willingness-to-Pay
CJRR: The Canadian Joint Replacement Registry
CADTH: Canadian Agency for Drugs and Technologies in Health
CHEERS: Consolidated Health Economic Evaluation Reporting Standards
VVC: Varus-Valgus Constraint
SD: Standard Deviations
(C): Total Costs
(E): Effectiveness
QALY: Quality-Adjusted Life Year
CEAC: Cost-Effectiveness Acceptability Curve
DSA: Deterministic Sensitivity Analyses
PSA: Probabilistic Sensitivity Analysis
ICE plane: Incremental Cost-Effectiveness Plane
