# Supplementary material for: Cost-effectiveness of total knee arthroplasty, unicompartmental knee arthroplasty, and high tibial osteotomy for medial compartment knee osteoarthritis in young patients: a Canadian public payer perspective
Source: J Orthop Surg Res. 2025 May 31;20:554. doi: 10.1186/s13018-025-05960-4 (PMC12125944; doi:10.1186/s13018-025-05960-4)
Supplement: Supplementary file 1 — Supplementary Material 1 [file 13018_2025_5960_MOESM1_ESM.docx]

**Supplementary Figure 1. The model’s performance against the prevalence of revision surgery and mortality rates post-total knee arthroplasty (TKA)**

**Supplementary Figure 2. The model’s performance against the prevalence of revision surgery and mortality rates post-unilateral knee arthroplasty (UKA)**

**Supplementary Figure 3. The model’s performance against the prevalence of revision surgery and mortality rates post-high tibial osteotomy (HTO)**

|  |
| --- |

**Supplementary Figure 1. The model’s performance against the prevalence of revision surgery and mortality rates post-total knee arthroplasty (TKA)**

**
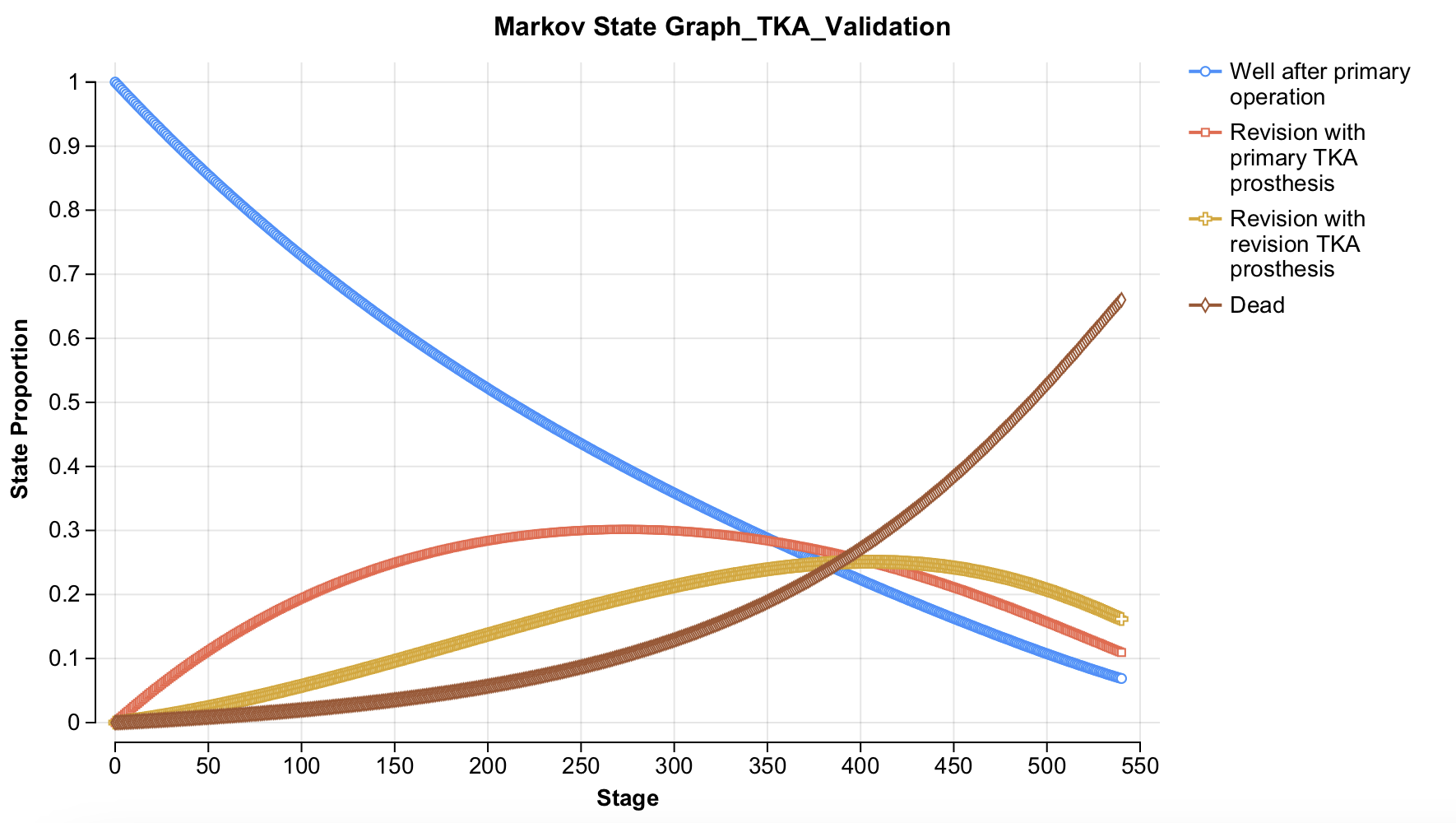
**

|  |
| --- |

**Supplementary Figure 2. The model’s performance against the prevalence of revision surgery and mortality rates post-unilateral knee arthroplasty (UKA)**

**
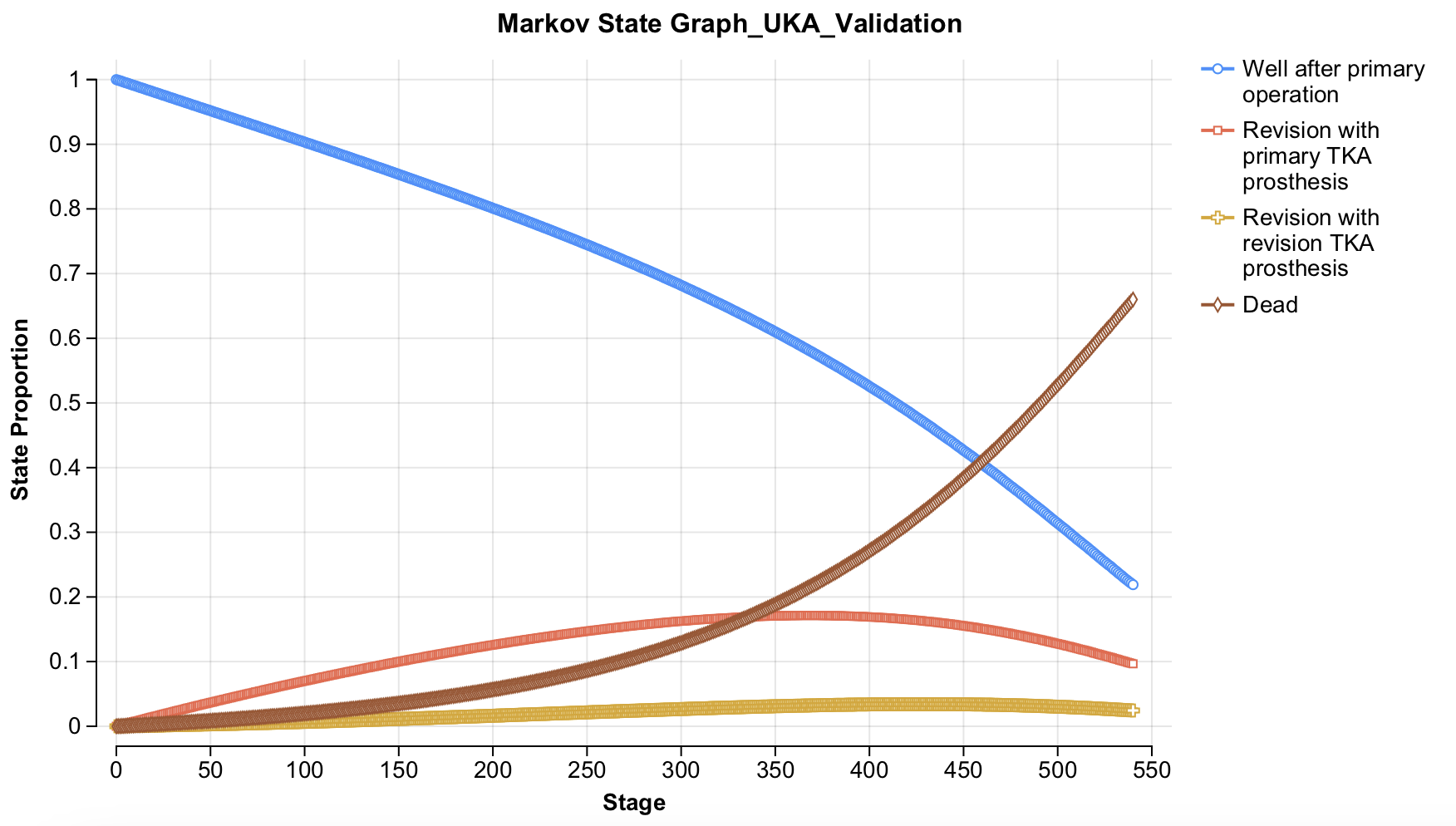
**

|  |
| --- |

**Supplementary Figure 3. The model’s performance against the prevalence of revision surgery and mortality rates post-high tibial osteotomy (HTO)**

**
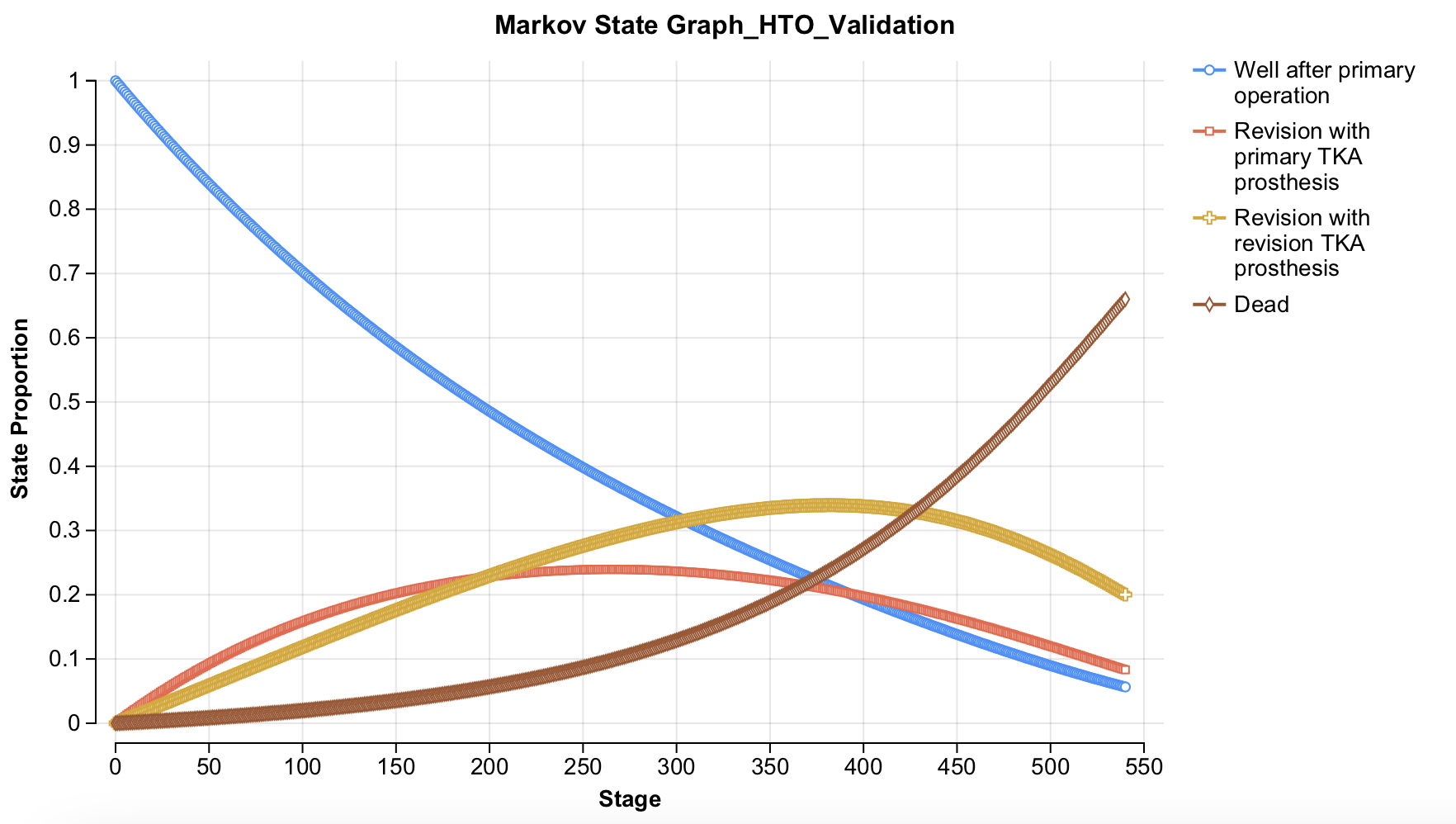
**
