# Supplementary material for: Cost-effectiveness of total knee arthroplasty, unicompartmental knee arthroplasty, and high tibial osteotomy for medial compartment knee osteoarthritis in young patients: a Canadian public payer perspective
Source: J Orthop Surg Res. 2025 May 31;20:554. doi: 10.1186/s13018-025-05960-4 (PMC12125944; doi:10.1186/s13018-025-05960-4)
Supplement: Supplementary file 3 — Supplementary Material 3 [file 13018_2025_5960_MOESM3_ESM.docx]

Supplementary Table 2. Canadian monthly probability of death by age group and sex.

| Age (years) | General  population | General lower bound | General upper bound | Male | Male lower bound | Male upper bound | Female | Female lower bound | Female upper bound |
| --- | --- | --- | --- | --- | --- | --- | --- | --- | --- |
| 45–46 | 0.0001526 | 0.0001221 | 0.0001831 | 0.0001994 | 0.0001595 | 0.0002393 | 0.0001092 | 8.74e-05 | 0.000131 |
| 46–47 | 0.0001626 | 0.0001301 | 0.0001951 | 0.0002119 | 0.0001695 | 0.0002543 | 0.0001167 | 9.34e-05 | 0.00014 |
| 47–48 | 0.0001743 | 0.0001394 | 0.0002092 | 0.0002253 | 0.0001802 | 0.0002704 | 0.0001251 | 0.0001001 | 0.0001501 |
| 48–49 | 0.0001869 | 0.0001495 | 0.0002243 | 0.0002412 | 0.000193 | 0.0002894 | 0.0001351 | 0.0001081 | 0.0001621 |
| 49–50 | 0.0002002 | 0.0001602 | 0.0002402 | 0.0002579 | 0.0002063 | 0.0003095 | 0.0001451 | 0.0001161 | 0.0001741 |
| 50–51 | 0.0002153 | 0.0001722 | 0.0002584 | 0.0002771 | 0.0002217 | 0.0003325 | 0.0001568 | 0.0001254 | 0.0001882 |
| 51–52 | 0.0002328 | 0.0001862 | 0.0002794 | 0.0002988 | 0.000239 | 0.0003586 | 0.0001693 | 0.0001354 | 0.0002032 |
| 52–53 | 0.0002512 | 0.000201 | 0.0003014 | 0.0003214 | 0.0002571 | 0.0003857 | 0.0001835 | 0.0001468 | 0.0002202 |
| 53–54 | 0.0002712 | 0.000217 | 0.0003254 | 0.0003465 | 0.0002772 | 0.0004158 | 0.0001994 | 0.0001595 | 0.0002393 |
| 54–55 | 0.0002938 | 0.000235 | 0.0003526 | 0.0003741 | 0.0002993 | 0.0004489 | 0.0002161 | 0.0001729 | 0.0002593 |
| 55–56 | 0.0003181 | 0.0002545 | 0.0003817 | 0.0004051 | 0.0003241 | 0.0004861 | 0.0002353 | 0.0001882 | 0.0002824 |
| 56–57 | 0.0003448 | 0.0002758 | 0.0004138 | 0.0004377 | 0.0003502 | 0.0005252 | 0.0002554 | 0.0002043 | 0.0003065 |
| 57–58 | 0.0003749 | 0.0002999 | 0.0004499 | 0.0004746 | 0.0003797 | 0.0005695 | 0.0002788 | 0.000223 | 0.0003346 |
| 58–59 | 0.0004076 | 0.0003261 | 0.0004891 | 0.0005148 | 0.0004118 | 0.0006178 | 0.0003038 | 0.000243 | 0.0003646 |
| 59–60 | 0.0004427 | 0.0003542 | 0.0005312 | 0.0005584 | 0.0004467 | 0.0006701 | 0.0003314 | 0.0002651 | 0.0003977 |
| 60–61 | 0.0004821 | 0.0003857 | 0.0005785 | 0.000607 | 0.0004856 | 0.0007284 | 0.0003624 | 0.0002899 | 0.0004349 |
| 61–62 | 0.0005257 | 0.0004206 | 0.0006308 | 0.0006599 | 0.0005279 | 0.0007919 | 0.0003967 | 0.0003174 | 0.000476 |
| 62–63 | 0.0005743 | 0.0004594 | 0.0006892 | 0.0007187 | 0.000575 | 0.0008624 | 0.0004344 | 0.0003475 | 0.0005213 |
| 63–64 | 0.0006272 | 0.0005018 | 0.0007526 | 0.0007842 | 0.0006274 | 0.000941 | 0.0004762 | 0.000381 | 0.0005714 |
| 64–65 | 0.0006859 | 0.0005487 | 0.0008231 | 0.0008557 | 0.0006846 | 0.0010268 | 0.0005232 | 0.0004186 | 0.0006278 |
| 65–66 | 0.0007506 | 0.0006005 | 0.0009007 | 0.0009348 | 0.0007478 | 0.0011218 | 0.0005751 | 0.0004601 | 0.0006901 |
| 66–67 | 0.0008229 | 0.0006583 | 0.0009875 | 0.0010224 | 0.0008179 | 0.0012269 | 0.0006339 | 0.0005071 | 0.0007607 |
| 67–68 | 0.0009028 | 0.0007222 | 0.0010834 | 0.0011202 | 0.0008962 | 0.0013442 | 0.0006985 | 0.0005588 | 0.0008382 |
| 68–69 | 0.0009921 | 0.0007937 | 0.0011905 | 0.0012274 | 0.0009819 | 0.0014729 | 0.0007708 | 0.0006166 | 0.000925 |
| 69–70 | 0.0010907 | 0.0008726 | 0.0013088 | 0.0013474 | 0.0010779 | 0.0016169 | 0.0008515 | 0.0006812 | 0.0010218 |
| 70–71 | 0.0012012 | 0.000961 | 0.0014414 | 0.0014803 | 0.0011842 | 0.0017764 | 0.0009424 | 0.0007539 | 0.0011309 |
| 71–72 | 0.0013238 | 0.001059 | 0.0015886 | 0.0016287 | 0.001303 | 0.0019544 | 0.0010435 | 0.0008348 | 0.0012522 |
| 72–73 | 0.00146 | 0.001168 | 0.001752 | 0.0017934 | 0.0014347 | 0.0021521 | 0.0011573 | 0.0009258 | 0.0013888 |
| 73–74 | 0.0016126 | 0.0012901 | 0.0019351 | 0.0019772 | 0.0015818 | 0.0023726 | 0.0012849 | 0.0010279 | 0.0015419 |
| 74–75 | 0.0017832 | 0.0014266 | 0.0021398 | 0.0021827 | 0.0017462 | 0.0026192 | 0.0014287 | 0.001143 | 0.0017144 |
| 75–76 | 0.0019746 | 0.0015797 | 0.0023695 | 0.0024126 | 0.0019301 | 0.0028951 | 0.0015905 | 0.0012724 | 0.0019086 |
| 76–77 | 0.0021887 | 0.001751 | 0.0026264 | 0.0026688 | 0.002135 | 0.0032026 | 0.001773 | 0.0014184 | 0.0021276 |
| 77–78 | 0.002428 | 0.0019424 | 0.0029136 | 0.0029568 | 0.0023654 | 0.0035482 | 0.001978 | 0.0015824 | 0.0023736 |
| 78–79 | 0.002698 | 0.0021584 | 0.0032376 | 0.0032793 | 0.0026234 | 0.0039352 | 0.0022109 | 0.0017687 | 0.0026531 |
| 79–80 | 0.0030007 | 0.0024006 | 0.0036008 | 0.0036421 | 0.0029137 | 0.0043705 | 0.0024742 | 0.0019794 | 0.002969 |
| 80–81 | 0.0033424 | 0.0026739 | 0.0040109 | 0.0040507 | 0.0032406 | 0.0048608 | 0.0027719 | 0.0022175 | 0.0033263 |
| 81–82 | 0.0037271 | 0.0029817 | 0.0044725 | 0.0045111 | 0.0036089 | 0.0054133 | 0.003111 | 0.0024888 | 0.0037332 |
| 82–83 | 0.0041623 | 0.0033298 | 0.0049948 | 0.0050302 | 0.0040242 | 0.0060362 | 0.0034956 | 0.0027965 | 0.0041947 |
| 83–84 | 0.004654 | 0.0037232 | 0.0055848 | 0.005617 | 0.0044936 | 0.0067404 | 0.0039331 | 0.0031465 | 0.0047197 |
| 84–85 | 0.0052109 | 0.0041687 | 0.0062531 | 0.0062817 | 0.0050254 | 0.007538 | 0.0044332 | 0.0035466 | 0.0053198 |
| 85–86 | 0.0058413 | 0.004673 | 0.0070096 | 0.0070365 | 0.0056292 | 0.0084438 | 0.0050059 | 0.0040047 | 0.0060071 |
| 86–87 | 0.0065562 | 0.005245 | 0.0078674 | 0.0078946 | 0.0063157 | 0.0094735 | 0.0056625 | 0.00453 | 0.006795 |
| 87–88 | 0.0073667 | 0.0058934 | 0.00884 | 0.0088711 | 0.0070969 | 0.0106453 | 0.0064141 | 0.0051313 | 0.0076969 |
| 88–89 | 0.0082845 | 0.0066276 | 0.0099414 | 0.0099835 | 0.0079868 | 0.0119802 | 0.0072738 | 0.005819 | 0.0087286 |
| 89–90 | 0.0093214 | 0.0074571 | 0.0111857 | 0.0112511 | 0.0090009 | 0.0135013 | 0.0082543 | 0.0066034 | 0.0099052 |
| 90–91 | 0.0104895 | 0.0083916 | 0.0125874 | 0.0126932 | 0.0101546 | 0.0152318 | 0.0093668 | 0.0074934 | 0.0112402 |
